# Serine Protease from *Nereis virens* Inhibits H1299 Lung Cancer Cell Proliferation via the PI3K/AKT/mTOR Pathway

**DOI:** 10.3390/md17060366

**Published:** 2019-06-20

**Authors:** Yanan Chen, Yunping Tang, Yanhua Tang, Zuisu Yang, Guofang Ding

**Affiliations:** 1 Zhejiang Provincial Engineering Technology Research Center of Marine Biomedical Products, School of Food and Pharmacy, Zhejiang Ocean University, Zhoushan 316022, China; cyanan2013@163.com (Y.C.); tyh19980126@163.com (Y.T.); abc1967@126.com (Z.Y.); 2 Zhejiang Marine Fisheries Research Institution, Zhoushan 316021, China

**Keywords:** lung cancer, nereis active protease, H1299 cells, PI3K/AKT/mTOR pathway

## Abstract

This study explores the in vitro anti-proliferative mechanism between Nereis Active Protease (NAP) and human lung cancer H1299 cells. Colony formation and migration of cells were significantly lowered, following NAP treatment. Flow cytometry results suggested that NAP-induced growth inhibition of H1299 cells is linked to apoptosis, and that NAP can arrest the cells at the G0/G1 phase. The ERK/MAPK and PI3K/AKT/mTOR pathways were selected for their RNA transcripts, and their roles in the anti-proliferative mechanism of NAP were studied using Western blots. Our results suggested that NAP led to the downregulation of p-ERK (Thr 202/Tyr 204), p-AKT (Ser 473), p-PI3K (p85), and p-mTOR (Ser 2448), suggesting that NAP-induced H1299 cell apoptosis occurs via the PI3K/AKT/mTOR pathway. Furthermore, specific inhibitors LY294002 and PD98059 were used to inhibit these two pathways. The effect of NAP on the downregulation of p-ERK and p-AKT was enhanced by the LY294002 (a PI3K inhibitor), while the inhibitor PD98059 had no obvious effect. Overall, the results suggested that NAP exhibits antiproliferative activity by inducing apoptosis, through the inhibition of the PI3K/AKT/mTOR pathway.

## 1. Introduction

Lung cancer is a highly malignant form of cancer that has some of the highest rates of morbidity and mortality worldwide [1,2]. Lung cancers caused the highest incidence of malignant tumors and led to 380,800 human deaths in Europe in 2018 [3]. Most lung cancer patients are in advanced stages at the time of diagnosis, which cannot be cured using surgery [4]. Currently, targeted drugs, such as tyrosine kinase inhibitors and immune checkpoint inhibitors, are used to treat cancers [5,6]. The advantage of targeted drugs is that they work primarily on cancer cells and have fewer side effects than chemotherapeutics. On the other hand, different cancer types exhibit different pathogenic behavior, and heterogeneity exists among cancer cells of the same type, so one targeted drug might be effective for one patient, but not for others [5,6]. Chemical drugs, such as cisplatin, carboplatin, and etoposide, are often used to treat advanced-stage lung cancers [7,8]. However, these drugs generally lead to cytotoxicity and side effects that limit their use as clinical treatments [9,10]. Therefore, improvements in the efficiency and toxicity profiles of anticancer drugs, are necessary for improving cancer treatments, which might be achievable through the use of natural products.

The earth’s surface is covered by 70% ocean; this is a unique environment that is a resource for new drug discovery [11,12]. In recent years, the research on marine organisms has expanded, and numerous substances with anti-cancer activities have been found [13]. *Nereis succinea*, for example, has been used to extract an abundance of materials for pharmacological research [14,15,16]. In addition, a decapeptide from *Perinereies aibuhitensis* was shown to exhibit anti-proliferative activity on human lung cancer H1299 cells [14]. Ge et al. [15] also found that a serine protease from *Neanthes japonica* exhibits anti-cancer activity toward leukemia cells. In our previous study, a serine protease from *Nereis virens* (Nereis Active Protease (NAP)) exhibited anti-proliferative activity toward human lung cancer cells, including A549, 95C, SPC-A-1, and H1299 cells [16], however, the mechanism underlying this remains unclear.

The PI3K/AKT/mTOR and ERK/MAPK pathways are often used to elucidate anti-tumor mechanisms [17,18,19,20,21]. The PI3K/AKT/mTOR pathway plays an important role in pathological processes, including cell differentiation, survival, and proliferation. Therefore, this pathway is considered as a major regulator of cancer progression [17]. Continuous activation of this pathway causes continuous cell growth that can lead to the evolution of cancer cells [9,18,22]. Since this is a gradual process, pan PI3K blockers, subtype-specific PI3K blockers, PI3K/mTOR double blockers, AKT blockers, and mTOR blockers have been developed to counteract the pathway’s influence on cancer formation [19]. In addition, the PI3K/AKT/mTOR pathway is connected to the ERK/MAPK pathway [20]. The activation of ERK is related to the continual growth of cells and affects the signal pathways related to cell proliferation. Previous studies suggest that apoptosis might be associated with the inhibition of the ERK/MAPK pathway [21,23,24]. Consequently, proteins in the PI3K/AKT/mTOR and ERK/MAPK signaling pathways could be good targets for cancer therapy. 

As the NAP exhibited the strongest anti-proliferative activity toward H1299 cells, in this study, transcriptome sequencing was first used to identify the significant signal pathways related to the treatment of H1299 cells with *N. virens* NAP. Furthermore, the PI3K/AKT/mTOR and ERK/MAPK pathways were chosen to explore the anti-proliferative mechanism of NAP on H1299 cells. This research indicated that NAP inhibits H1299 cell proliferation via the PI3K/AKT/mTOR pathway. Therefore, NAP from *N. virens* demonstrates a strong potential as an anti-lung cancer drug candidate. 

## 2. Results and Discussion

### 2.1. NAP Inhibits the Growth and Migration of H1299 Cells

**Figure 1:**
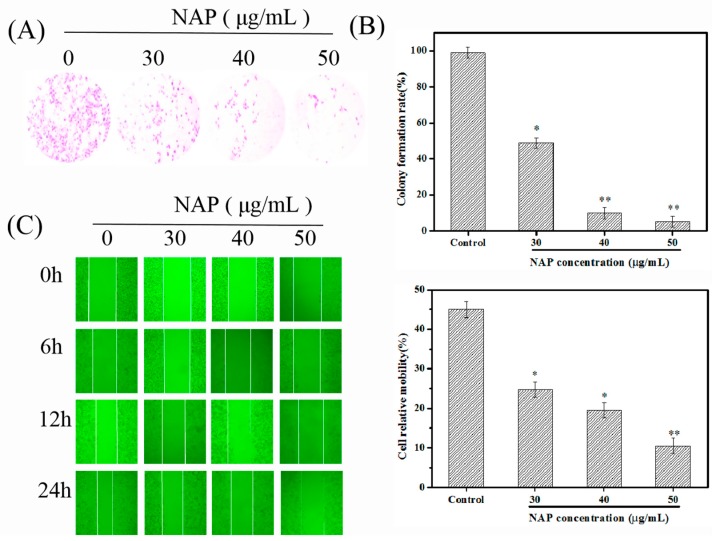
Nereis Active Protease (NAP)-based inhibition of the growth and migration of H1299 cells. (**A**) H1299 cells were treated with NAP (0, 30, 40, and 50 μg/mL) for 24 h and then cultured in RPMI-1640 complete culture medium for 12 days, to investigate the ability of single cells to form colonies through a colony formation assay. Magnification: 100 ×. (**B**) Colony formation rate of H1299 cells. (**C**) H1299 cells were treated with NAP (0, 30, 40, and 50 μg/mL) for 24 h and photographed under a microscope at 0, 6, 12, and 24 h. Magnification: 100 ×. (**D**) Wound healing rates after treatment of H1299 cells with NAP for one day. Significant results: * *p* ≤ 0.05; ** *p* ≤ 0.01 vs the blank group (0 μg/mL NAP).

Malignant cell proliferation is an uncontrolled process that increases the risk of carcinogenic factors that facilitate the dispersion and migration of cancer cells [25]. The inhibition of cancer cell growth and migration are effective ways to control tumor development [25]. In this work, the influence of NAP on the proliferation of individual H1299 cells was studied using a colony formation assay. The results indicated that the colony formation rate of H1299 cells significantly decreased after the NAP treatment (Figure 1A,B). The results were consistent with our previous studies [16], indicating that NAP could significantly inhibit the growth and proliferation of H1299 cells. Furthermore, a scratch wound assay was used to investigate the influence of NAP on the migrative ability of H1299 cells. Results revealed that NAP could inhibit wound healing through the inhibition of H1299 cell migration, after 24 h of treatment (Figure 1C,D). A similar phenomenon was reported by Song et al. [26], who found that a serine protease (*Trichosanthes kirilowii*) inhibited the proliferation of colorectal cancer cells. 

### 2.2. NAP-Induced G0/G1 Phase Block in H1299 Cells

In the process of normal cell growth and proliferation, the cell cycle is divided into G0/G1, S and G2/M stages. G1 to S is a particularly important stage in the cell cycle [27]. During the period of complex and active molecular level changes, DNA replication is regulated by cyclin-dependent kinases (CDK), and cyclin D, and cyclin E proteins, which are easily affected by environmental conditions [28]. The regulation of G1 to S is thought to be of great significance for controlling the growth of tumors [29].

**Figure 2:**
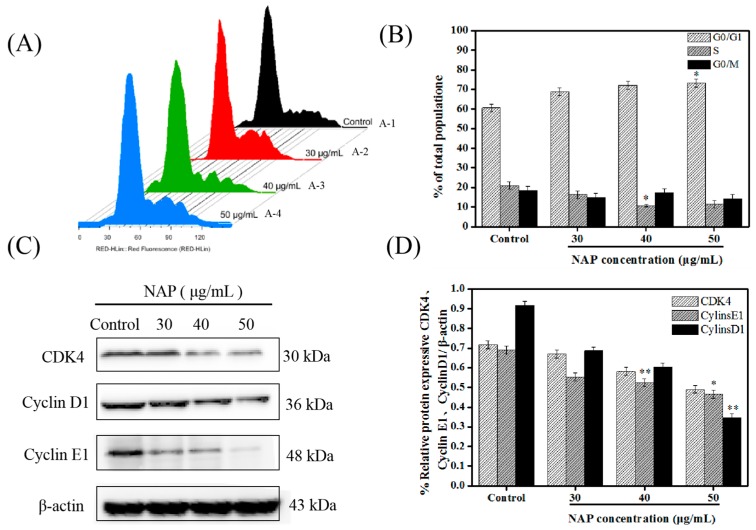
Flow cytometry of the cell cycle phase distribution reveals a NAP-induced G0/G1 stage block in H1299 cells. (**A**) Percentage of H1299 cells at each phase A1—control group; A2—30 μM NAP-treated group; A3—40 μg/mL NAP-treated group, and A4—50 μg/mL NAP-treated group. (**B**) Percentage of NAP-treated H1299 cells at the three stages of the cell cycle. (**C**) Western blot measurements of the cyclin-related proteins. (**D**) Ratio of CDK 4/β-actin, cyclin D1/β-actin, and cyclin E1/β-actin values. * *p* ≤ 0.05, ** *p* ≤ 0.01 versus cells without NAP treatment.

Flow cytometry was applied for subsequent investigation of the influence of NAP on the cell cycle. The percentages of cells blocked by NAP (0, 30, 40 and 50 μg/mL) at the G0/G1 phase were 60.7% ± 1.8, 68.9 ± 2.1%, 72.0 ± 1.9%, and 74.3 ± 1.5%, respectively (Figure 2A,B). In addition, the expression levels of CDK4, cyclin E1 and cyclin D1 proteins in the G0/G1 phase were examined [30,31]. Western blot results showed that CDK4, cyclin E1, and cyclin D1 proteins were down-regulated, indicating that NAP could have induced apoptosis by blocking the G0/G1 phase in H1299 cells (Figure 2C,D). These results were supported by results from Han et al. [30] who found that 8-Cetylcoptisine blocked A549 cells in the G0/G1 phase, and Zhang et al. [32] who demonstrated that the up-regulation of MiR-101-3p could significantly reduce the viability of H1299 cells and that MiR-101-3p up-regulation could block the cell cycle in the G0/G1 phase of H1299 cells.

### 2.3. Influence of NAP on the Transcriptome of H1299 Cells

**Figure 3:**
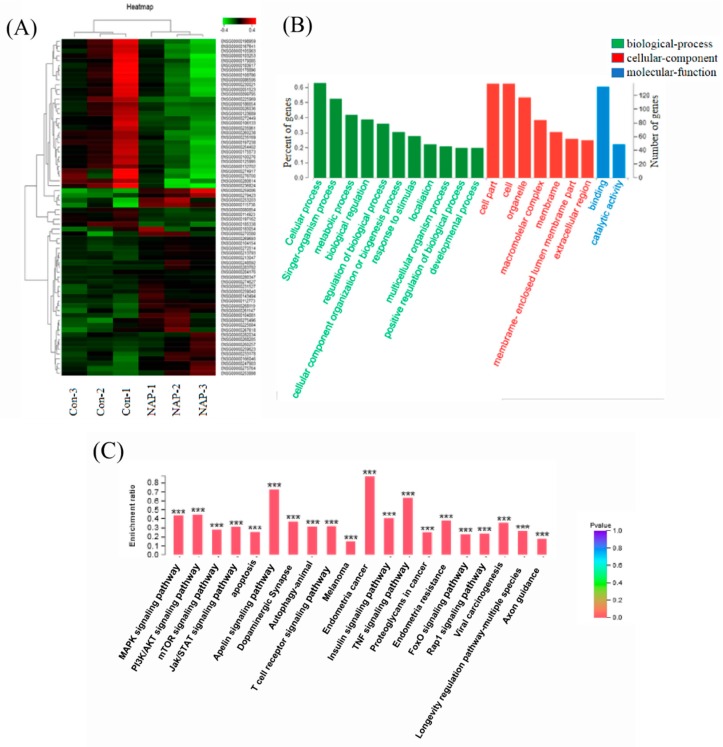
Influence of NAP on the transcriptome of H1299 cells. (**A**) Heat-map for 219 significantly different genes between the NAP-treated (NAP 1–3) and the control cells (Con 1–3). (**B**) Differential genes annotated according to the gene ontology (GO) classification criteria. (**C**) Annotations according to the enrichment of pathways and significant enrichment of 20 pathways.

Transcriptome sequencing is a major method used for studying gene expression. This method can highlight significant differentially expressed genes, which can be used to determine the major signaling pathways involved in biological processes [33]. In the process of RNA sequencing, a large concentration of cells was required. When the concentration of NAP is more than 30 μg/mL, cells will apoptosis and suspend in the medium. This is not conducive to collecting a large numbers of cells. Therefore, in the present study, 30 μg/mL of NAP was chosen for the transcriptome analysis. However, mRNA from H1299 cells with and without NAP treatment, was extracted. An mRNA library for the H1299 cells was constructed and a paired *t*-test was performed on three balanced experiments, with a *p*-value < 0.01 set as the significance level. Two hundred and nineteen differentially expressed genes were detected between the NAP-treated H1299 cells and the control group (Figure 3). Logarithmic ratios and *p*-values of the 219 genes were used to detect up- and down-regulated signaling pathways after the NAP treatment. Enrichment analysis of the KEGG pathways was performed for the 219 differentially expressed genes. Pathways with significant differences in pathway enrichment were selected for further study.

PI3K/AKT/mTOR and ERK/MAPK pathways are often related to the growth and proliferation of cancer cells [8,34,35]. For example, Li et al. [36] studied Tyr-Val-Pro-Gly-Pro (AAP-H), an active peptide extracted and purified from Anthopleura anjunae, which induces apoptosis in prostate cancer DU145 cells, via the PI3K/AKT/mTOR pathway. Liu et al. [37] demonstrated that 1, 4-naphthoquinone induces apoptosis in lung cancer cells by activating oxygen-dependent down-regulation of the proteins related to the MAPK cascade. Therefore, these two pathways were selected for investigation, for their role in NAP-induced apoptosis of H1299 cells.

### 2.4. Influence of NAP on the ERK/MAPK Pathway in H1299 Cells

Previous experiments have shown that the inhibitory activity of NAP on the H1299 cells is related to regulation of the cell cycle and the induction of apoptosis [16]. Transcriptome sequencing results have indicated that the PI3K/AKT/mTOR and ERK/MAPK pathways are associated with NAP’s inhibitory effect on the H1299 cells. Johnson et al. [38] found that MAPK is closely related to the initiation of apoptosis and cell cycle quiescence, in various tumor cell lines. Therefore, the influence of NAP on the phosphorylation of the MAPK pathway-related kinases was investigated through Western blotting. 

**Figure 4:**
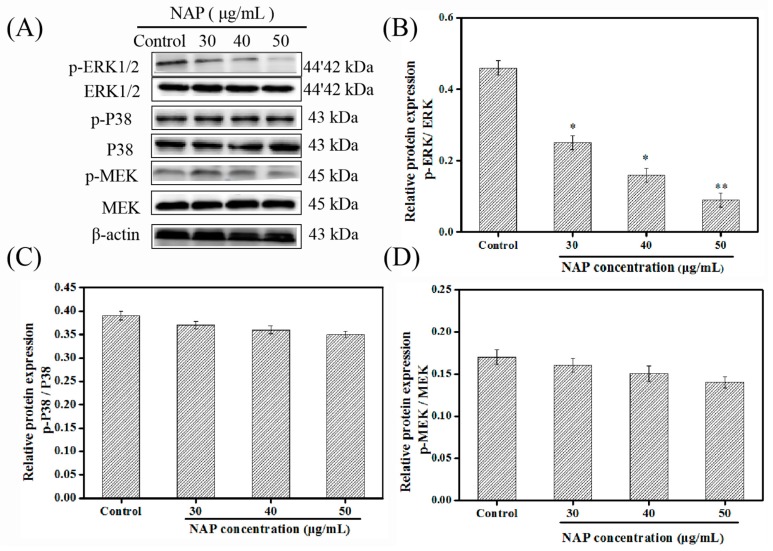
Influence of NAP (0, 30, 40 and 50 μg/mL) on the ERK/MAPK cascade in H1299 cells. (**A**) Western blot visualizations of the ERK/MAPK pathway-related proteins from the NAP-treated H1299 cells. (**B**) The ratio of phosphorylated ERK (p-ERK) to ERK in the NAP-treated H1299 cells. (**C**) The ratio of phosphorylated P38 (p-P38) to P38 in NAP-treated H1299 cells. (**D**) The ratio of phosphorylation MEK (p-MEK) to MEK in the NAP-treated H1299 cells. * *p* ≤ 0.05, ** *p* ≤ 0.01 versus cells without NAP treatment.

H1299 cells were treated with NAP and Western blots were used to visualize and measure ERK/MAPK pathway-related proteins (Figure 4). Results indicated that NAP treatment might have led to down-regulated p-ERK levels in H1299 cells, and the expression of p-P38 and p-MEK protein did not change significantly, when compared to cells without NAP treatment. Importantly, by inhibiting the activation of the PI3K/AKT/mTOR pathway, ERK phosphorylation might have been inhibited [39]. Therefore, we speculated that NAP might inhibit ERK phosphorylation by inhibiting the PI3K/AKT/mTOR pathway.

### 2.5. Influence of NAP on the PI3K/AKT/mTOR Pathway in H1299 Cells

The PI3K/AKT/mTOR pathway could activate or inhibit ERK, which has the same effect on cell proliferation and apoptosis [40]. The growth, proliferation, differentiation, and death of cells are regulated by PI3K and AKT protein kinases [41]. Research has indicated that this pathway plays a critical role in the abnormal activation of tumor cells [42]. When PI3K is activated, AKT is activated downstream through phosphorylation, due to the exposure of its phosphorylation site; this leads to the downstream activation of mTOR and other proteins, which plays an anti-apoptotic role within the cell [43,44].

**Figure 5:**
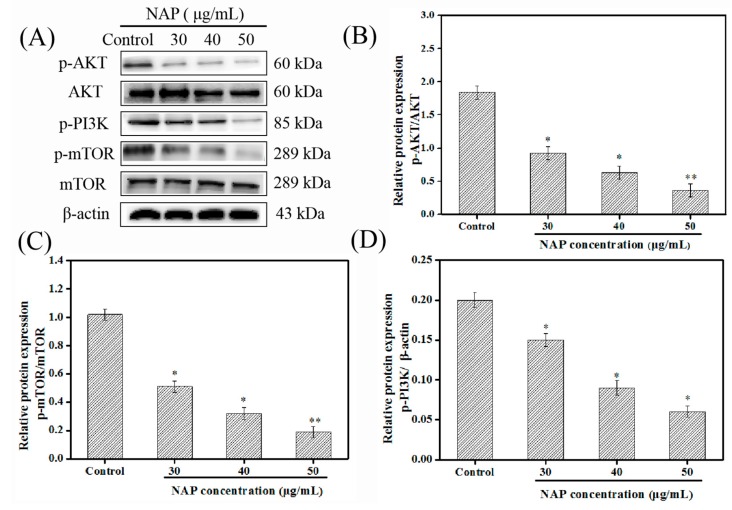
Influence of the NAP on PI3K/AKT/mTOR cascade pathway in H1299 cells. (**A**) Western blot visualizations of PI3K/AKT/mTOR pathway-related proteins from the NAP-treated H1299 cells. (**B**) The ratio of phosphorylated AKT (p-AKT) to AKT in NAP-treated H1299 cells. (**C**) The ratio of phosphorylated mTOR (p-mTOR) to mTOR, in the NAP-treated H1299 cells (**D**) The phosphorylation ratio of PI3K (p-PI3K) in the NAP-treated H1299 cells. * *p* ≤ 0.05, ** *p* ≤ 0.01 versus cells without NAP treatment.

To determine the effect of NAP concentrations (0, 30, 40, and 50 μg/mL) on the PI3K/AKT/mTOR pathway, PI3K, AKT, and mTOR were chosen for study, visualized, and analyzed, using Western blots (Figure 5). Results indicated that the NAP treatment led to the downregulation of p-mTOR, p-AKT, and p-PI3K levels in the H1299 cells, while PI3K, AKT, and mTOR levels did not change. Therefore, this suggests that the role of NAP in promoting apoptosis might be mediated via the inhibition of the PI3K/AKT/mTOR pathway in H1299 cells.

### 2.6. NAP-Induced H1299 Cell Apoptosis Involves the PI3K/AKT/mTOR and ERK/MAPK Pathways

**Figure 6:**
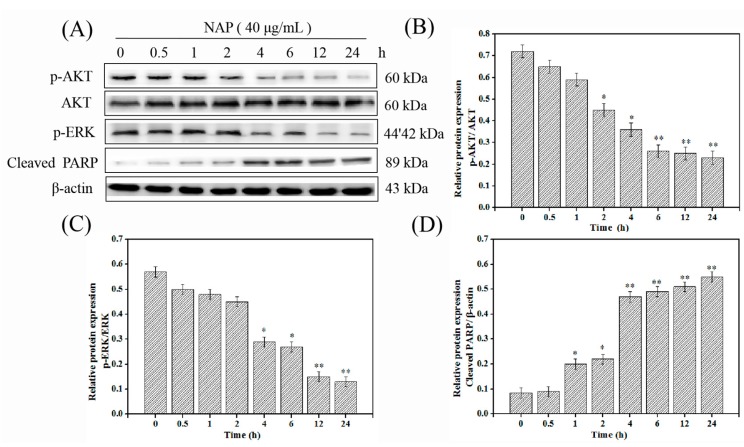
Influence of NAP on PI3K/AKT/mTOR and ERK/MAPK cascade at different time points. (**A**) Expression of p-AKT, AKT, p-ERK, and cleaved PARP proteins in H1299 cells treated with NAP at different time points. (**B**) The ratio of p-AKT (Ser473)/AKT in NAP-treated H1299 cells. (**C**) The ratio of p-ERK (Thr202/Tyr204)/β-actin in NAP-treated H1299 cells. (**D**) The ratio of cleaved-PARP/β-actin in NAP-treated H1299 cells. * *p* ≤ 0.05, ** *p* ≤ 0.01 versus cells without NAP treatment.

A further investigation was carried out to verify if the NAP-induced apoptosis is closely associated with the PI3K/AKT/mTOR and ERK/MAPK pathways in the H1299 cells. Cells were treated with 40 μg/mL NAP, then tested for changes in p-ERK, p-AKT, and cleaved PARP proteins, at different times [26,36]. The results indicated that NAP treatment led to the downregulation of p-AKT and p-ERK levels, and upregulation of the levels of cleaved-PARP (Figure 6). Importantly, the cleaved-PARP levels began to increase with concurrent decreases in levels of phosphorylated ERK and AKT. Results indicated that NAP could induce the apoptosis of H1299 cells via the PI3K/AKT/mTOR and ERK/MAPK pathways.

**Figure 7:**
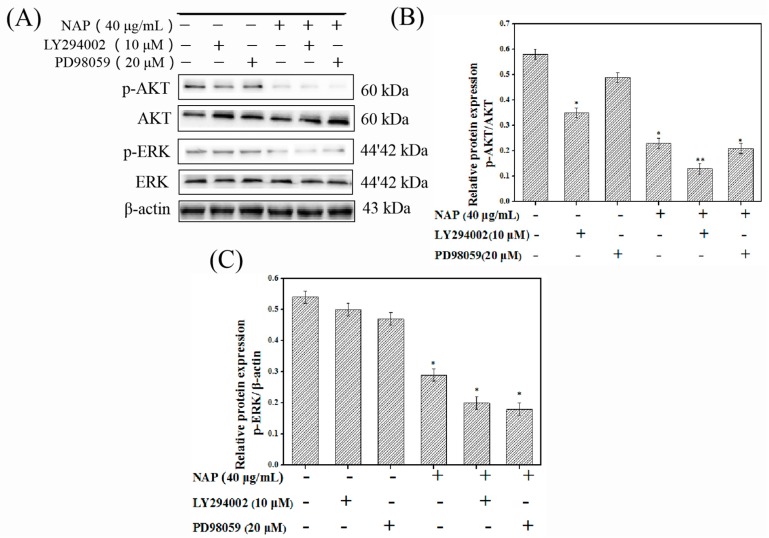
Influence of LY294002 and PD98059 on the PI3K/AKT/mTOR and ERK/MAPK pathway-related proteins. (**A**) Influence of LY294002 and PD98059 on ERK, AKT, p-ERK, and p-AKT proteins in H1299 cells. (**B**) p-AKT/AKT ratio expressed in H1299 cells. (**C**) Expression of p-ERK ratio expressed in H1299 cells.

The inhibitors LY294002 and PD98059 were also applied to the cells, to inhibit these two pathways. Their effects on the NAP-induced apoptosis were assessed by Western blot analysis. Results indicated that treatment with LY294002 enhanced the NAP’s effects on p-ERK and p-AKT proteins, while treatment with PD98059 did not enhance the NAP’s effects on p-ERK and p-AKT proteins (Figure 7A–C). Therefore, our studies suggested that NAP could induce H1299 cell apoptosis by down-regulating the related proteins in the PI3K/AKT/mTOR pathway.

## 3. Materials and Methods

### 3.1. Cell Culture

The H1299 cell lines were purchased from the Cell Bank of Chinese Academy of Sciences (Shanghai, China). The cells were incubated in RPMI-1640 medium containing 10% FBS, 1% 100 U/mL penicillin and streptomycin, and incubated in an incubator (Forma 3111, Thermo, Waltham, MA, USA) containing 5% CO_2_ at 37 °C.

### 3.2. Material Sources

NAP was purified from *N. virens* and stored in our laboratory [16,45]. Fetal bovine serum (FBS) without mycoplasma, bacteriophage, and low endotoxin was purchased from Zhejiang Tianhang Biotechnology Co., Ltd. (Hangzhou, China). Antibodies against AKT, ERK, P38, MEK, mTOR, PARP, p-AKT, p-PI3K, p-mTOR, p-ERK, p-P38, p-MEK, cyclin B1, PARP, CDK4, and cyclin D1, were purchased from the Cell Signaling Technology (Boston, MA, USA). A cell cycle analysis kit was obtained from BestBio Science Co., (Shanghai, China). 

### 3.3. Clonogenic Survival Assay

A clonogenic survival assay was applied for detecting single-cell survival, after treatment with NAP [46]. For adherent cells, 500 single cell suspensions of H1299 cells were inoculated into 6-well plates. After adherence, NAP (0, 30, 40, and 50 μg/mL) was added and removed after 24 h. Cells were then cultured in RPMI-1640 complete medium for 12 days, and the medium was washed over with PBS. Poly-formaldehyde liquid (4%) was applied for approximately 15 min to fix the cells, and the cells were then stained with 1% crystal violet for 15 min at 25 °C. The 6-well plates were rinsed gently with water and the number of clones (more than 50 cells) were determined using a microscope (OLYMPUS, Tokyo, Japan). Clone formation rate = (colonies/seed cells) × 100%.

### 3.4. Cell Migration (Scratch Wound) Assay

The migration of cells after the NAP-treatment was tested by wound scratch assay [47]. H1299 cells were inoculated in a 6-well culture plate and the appropriate medium was added until the cells covered 80% of the plate’s surface, then, the cells were scraped to form a “wound” with a sterilized pipette tip. The 6-well plates were then washed twice with PBS, and photographed at 0, 6, 12, and 24 h, respectively. Finally, Image J software (NIH, Bethesda, MD, USA) was used to calculate the wound area.

$$Relative\;mobility\;\left(\%\right)\;=\;\frac{\left(Wound\;area_{\left(0\;h\right)}\;-\;Wound\;area_{\left(24\;h\right)}\right)}{Wound\;area_{\left(0\;h\right)}\;}\;\times \;100$$

### 3.5. Cell Cycle Experiment 

A cell cycle arrest test was used to determine the proportion of cells in different cell cycle phases after treatment with NAP [48]. Cells were inoculated in 6-well culture plates, and incubated until the cells covered 70% of the plate’s surface. Then, the cells were treated with NAP (0, 30, 40, and 50 μg/mL) for 24 h, respectively. The steps for cell cycle arrest tests were in accordance with the method described by Li et al. [36]. Finally, flow cytometry was used to detect the cell cycle phase.

### 3.6. RNA Extraction and Integrity Test

H1299 cells in the log-phase growth were digested and dispersed to form single cells using trypsin, then incubated in an incubator for 24 h, treated with 0 μg/mL (blank control group), and 30 μg/mL NAP (medication group), for 24 h, and washed with PBS. The cells were lysed in 1 mL trizol solution to extract the total RNA. Transcriptome sequencing was performed by Sangon Biotech (Shanghai, China).

### 3.7. Western Blotting

The steps of Western blotting were in accordance with a method described by Li et al. [36]. A total of 40 μg of the samples were used for detection, in this study. Western blot membranes were probed with an enhanced chemiluminescence antibody and the images were obtained using an Alpha FluorChem FC3 imaging system (ProteinSimple, San Jose, CA, USA). The level of protein expression was quantified using Image J software. β-actin was used as the control.

### 3.8. Statistical Analysis

SPSS 19.0 software was used to conduct one-way ANOVA on the data; the results represent the mean with the standard deviation (*n* = 3). A *p*-value of ≤ 0.05 was considered to show a statistically significant difference between the two groups.

## 4. Conclusions

This research showed that NAP from *N. virens* exhibits strong anti-proliferative activity through the inhibition of proliferation and migration, and by blocking H1299 cells in the G0/G1 phase. Further studies showed that NAP exhibits anti-proliferative activity against human lung cancer H1299 cells, by inhibiting the PI3K/AKT/mTOR pathway. Therefore, NAP shows promising results that highlight its potential for treating lung cancer in the future.
